# Impact of negative inotropic drugs on accuracy of diastolic stress echocardiography for evaluation of left ventricular filling pressure

**DOI:** 10.1038/s41598-017-10301-5

**Published:** 2017-08-25

**Authors:** Joe-Elie Salem, Florent Laveau, Alexandre Ceccaldi, Christian Funck-Brentano, Jean Sebastien Hulot, Amel Mameri, Olivier Barthelemy, Gerard Helft, Claude Le Feuvre, Richard Isnard, Nadjib Hammoudi

**Affiliations:** Sorbonne Universités, UPMC Univ Paris 06, AP-HP, CIC 1421, CIC-Paris Est, Cardiology department-Echocardiography Unit, Pharmacology department, INSERM-U1166 ICAN, Pitié-Salpêtrière Hospital, F-75013 Paris, France

## Abstract

The ratio of early diastolic trans-mitral flow velocity to tissue-Doppler mitral annular early diastolic velocity (E/e′), and left ventricular end-diastolic pressure(LVEDP) have been shown to be correlated at rest, provided that patients are not on positive inotropic drugs. Data concerning the latter correlation during exercise stress are conflicting. Therefore, we investigated if use of negative inotropic drugs (NID), impacts the accuracy of E/e′ as a surrogate for LVEDP during low-level exercise. An exercise(50 watts) during cardiac invasive hemodynamic monitoring and an exercise echocardiography were performed prospectively within 24 hours in 54 patients (81%male, 62 ± 9years) with preserved LV Ejection-Fraction. Before exercise, the patients had scattered LVEDP (13.8 ± 5.8 mmHg) and septal E/e′ (8.7 ± 2.7). Half of them were on NID, mainly betablockers(n = 26). The correlation between septal-E/e′ and LVEDP was low for examinations performed at rest (r = 0.35,p = 0.01) with no significant impact of NID. For measurements performed at 50 Watts, NID had a significant impact on the association between septal-E/e′50 watts and LVEDP50 watts (β = −0.28,p = 0.03). Correlation between septal-E/e′50 watts and LVEDP50 watts persisted in patients on NID (r = 0.61,p = 0.001) while it disappeared in the group of patients with no NID (r = 0.15,p = 0.47). NID use is an important confounding factor to take into consideration when assessing exercise LVFP using stress E/e′ in patients with preserved LVEF.

## Introduction

Cardiac catheterization has demonstrated its usefulness for the diagnosis of early stage of heart failure with preserved ejection fraction (HFpEF), which is characterized by exercise-induced abnormal left ventricular filling pressure (LVFP) despite normal resting values^1, 2^. However, the invasive nature of this exploration limits its dissemination and echocardiography has been proposed as an alternative method for LVFP monitoring^3, 4^. The ratio of early diastolic trans-mitral flow velocity to tissue-Doppler mitral annular early diastolic velocity (E/e′) is currently the most widely used Doppler ultrasound parameter to diagnose elevation of LVFP. Several studies have shown a good correlation between E/e′ and LVFP at rest, in patients with chronic heart failure, either with preserved or altered left ventricular ejection fraction (LVEF)^5–8^. However, recent data have challenged the validity of this parameter^9, 10^. In particular, the use of E/e′ during exercise is a matter of debate^3, 11–14^. Stress E/e′ has first been validated in a study of small sample size, in patients with heterogeneous LVEF almost all treated with beta-blockers^3^. However, conflicting results have been reported in other studies assessing E/e′ to evaluate exercise LVFP in various populations with preserved LVEF, including untreated healthy subjects^11, 13, 15^.

Pulsed-Doppler early diastolic transmitral peak flow velocity (E) is considered to be a composite parameter mainly depending on LVFP and LV relaxation^16^. Nagueh *et al*., suggested using the ratio of E to tissue-Doppler mitral annular early diastolic velocity, E/e′, as a surrogate for invasive LVFP. E was estimated to be corrected for the influence of LV relaxation by e′, which was considered a preload-independent index of LV relaxation^17^. Positive and negative inotropic drugs have been shown to modify LV relaxation^18–20^ and also affect e′ preload-independence in experimental conditions^18^. In a canine model, when LV relaxation was impaired by beta-blockers use (Tau constant > 65 msec), e′ was preload-independent. In contrast, when dobutamine was used, LV relaxation was enhanced (Tau constant < 50 msec) and e′ lost its preload-independence^18^. Confirming these non-clinical data, our group and others reported that positive inotropic agents severely impaired the correlation between E/e′ and invasive evaluation of LVFP in patients with decompensated end-stage systolic heart failure^21, 22^ while this correlation was good in the same type of patients treated by beta-blockers^6^. We therefore hypothesized that exercise, by releasing endogenous catecholamine^23^, also alters the correlation between E/e′ and LVFP during stress echocardiography unless patients are concomitantly treated with negative inotropic drugs (NID) such as beta-blockers. In order to test this hypothesis, the aim of this study was to assess the impact of NID use on accuracy of stress E/e′ as a surrogate for exercise LVFP using a direct invasive measurement as the reference method in patients at high risk of heart failure with preserved ejection fraction (HFpEF).

## Results

### Clinical and demographic characteristics

Among the 60 patients initially enrolled in the study, 6 patients had incomplete data during exercise (missing E/e′ for 2; missing hemodynamic data for 4); leaving 54 patients for final analysis. Patients were mainly male (81%) with a mean age of 62 ± 9 years. Sixty-one, 28 and 39% of patients had hypertension, diabetes or history of coronaropathy, respectively. Twenty-seven patients (50%) were treated by a NID, mainly a beta-blocker (n = 26), whose type and doses are detailed in Table 1. One patient received verapamil. Baseline clinical data did not significantly differ between patients with and without NID (Table 2). Previous history of coronaropathy tended to be more frequent in NID vs. no NID subgroups (52% vs 26%, p = 0.09). There was no difference between systolic arterial pressure measured during echocardiography and catheterization at rest (138 ± 19 vs. 140 ± 20 mmHg, ns; respectively), 25 watts (155 ± 21 vs. 161 ± 19 mmHg, ns; respectively) and 50 watts (169 ± 23 vs. 169 ± 24 mmHg, ns; respectively).

**Table 1:** Negative inotropic drug used at the time of LVEDP evaluation (n = 27).

Type of negative inotropic agent	Dose of (mg/day)
**Beta-blockers** 26 (46)	
- Bisoprolol 10 (19)	5 [2.5–10]
- Acebutolol 7 (13)	200 [150–200]
- Atenolol 5 (9)	100 [50–100]
- Nebivolol 2 (4)	2.5–5
- Celiprolol 1 (2)	200
- Sotalol 1 (2)	320
**Calcium channel blocker** 1 (4)	
- Verapamil	120

The values given are n (%) or medians ± IQR.

**Table 2:** Demographic, clinical, biological and invasive hemodynamic data of analyzed patients.

	No NID	NID	p
Number of patients	27	27	
**Demographic characteristics**			
Male	22 (81%)	22 (81%)	1
Age (Years)	63 ± 9	61 ± 9	0.42
Body surface area (m²)	1.9 ± 0.3	1.9 ± 0.2	1
Hypertension	14 (52%)	19 (70%)	0.26
Diabetes	7 (26%)	8 (29%)	0.18
Dyslipidaemia	18 (67%)	18 (67%)	1
Ischemic cardiomyopathy	7 (26%)	14 (52%)	0.09
RAA antagonist (≥1 drug)	13 (48%)	14 (52%)	1
Dihydropyridine	4 (15%)	7 (26%)	0.5
Insulin	2 (7%)	1 (4%)	1
**Biological**			
Creatinine (µmol/L)	85 ± 22	88 ± 21	0.61
Hemoglobin (g/dL)	14.5 ± 1.2	14.1 ± 1.3	0.25
**Hemodynamics**			
LVEDP at rest (mmHg)	13 ± 5.6	14.7 ± 6	0.29
LVEDP at 25 Watts (mmHg)	19.9 ± 7.2	23.1 ± 7.6	0.12
LVEDP at 50 Watts (mmHg)	21.7 ± 8.4	26.4 ± 7.2*	0.03
LVEDP > 16 mmHg at rest	5 (19%)	8 (29%)	0.53
LVEDP > 16 mmHg during exercise	19 (70%)	24 (89%)	0.18
LVEDP ≥ 25 mmHg during exercise	14 (52%)	17 (63%)	0.58
LVSP at rest (mmHg)	131 ± 20	131 ± 20	1
LVSP at 25 Watts (mmHg)	159 ± 26	160 ± 21	0.88
LVSP at 50 Watts (mmHg)	167 ± 26	170 ± 21	0.64
**Clinical characteristics**			
Overt heart failure history	0 (0%)	0 (0%)	1
Dyspnea during stress exercise _50 Watts_	11 (41%)	11 (41%)	1

Values are means ± SD or *n* (%). Abbreviations: LVEDP: Left-ventricular end-diastolic pressure; LVSP: Left-ventricular systolic pressure; NID: Negative inotropic drug; RAA: renin-angiotensin-aldosterone antagonists (i.e angiotensin-converting enzyme inhibitors, angiotensin II and aldosterone receptor blockers).

### Echocardiography and catheterization results

#### Invasive hemodynamic monitoring data

**Figure 1:**
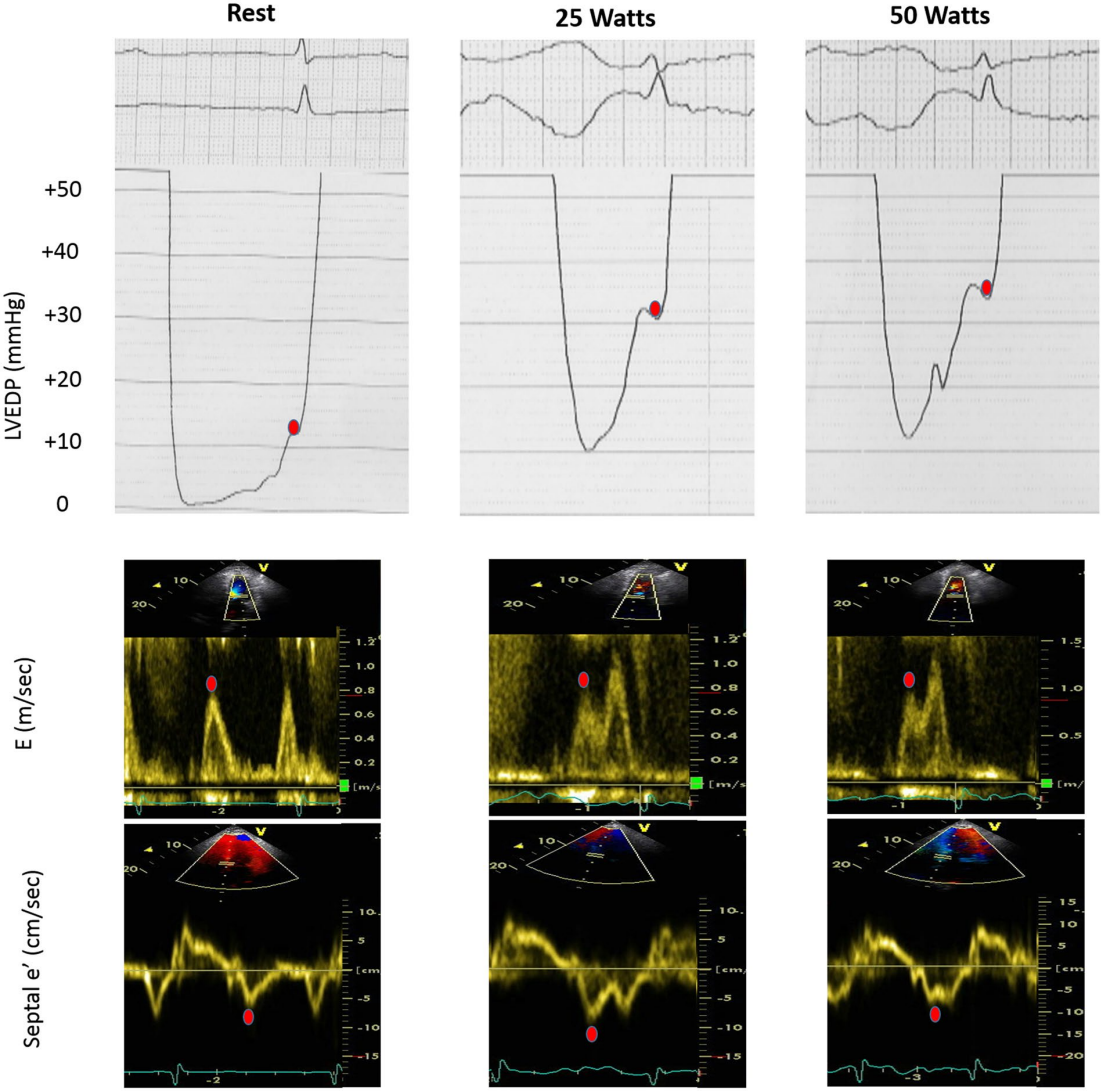
Example of Doppler and hemodynamic recordings for a patient included in this study with parameters at rest, 25 Watts and 50 Watts.

An example of data acquisition is presented in Fig. 1. Detailed results are provided in Table 2. Our patients had scattered baseline LVEDP at rest (13.8 ± 5.8 mmHg), with 24% having LVEDP >16 mmHg at rest. LVEDP increased at 25 Watts (21.5 ± 7.5 mmHg) and 50 Watts (24 ± 8.1 mmHg), as compared to LVEDP at rest (p < 0.001). During exercise, 57% of all patients had an LVEDP ≥ 25 mmHg, without any influence of NID use (52% in patients without NID and 63% in NID patients, p = 0.58). A total of 41% experienced dyspnea at 50 watts, with no association with NID use (Table 2). LVEDP and heart rate were not significantly associated at any level of the hemodynamic evaluations in subgroups of patients with NID and without NID.

#### Echocardiography data

The median time between echocardiography and catheterization was 4 hours 49 minutes. There were no significant differences in baseline echocardiographic data according to NID use or not (Table 3). No patient had mitral regurgitation >grade 1. Main diastolic echocardiographic parameters at 25 and 50 watts in both groups are summarized in Table 3. E, lateral and septal e′, and their respective E/e′ values were not significantly different between NID users and non-users at any exercise level. The heart rate tended to be lower in patients with vs. without NID at rest (62.6 ± 11.1 vs. 66.4 ± 9.8bpm, p = 0.19), at 25 watts (82.6 ± 11.8 vs. 88.4 ± 10.5bpm, p = 0.06) and at 50 watts (93.1 ± 11.3 vs. 98.2 ± 12.3bpm, p = 0.12).

**Table 3:** Echocardiographic data at the time of LVFP evaluation.

	No NID	NID	p
Number of examinations	27	27	
**Data Baseline**			
Heart rate (bpm)	66.4 ± 9.8	62.6 ± 11.1	0.19
Left ventricular tele-diastolic diameter (mm)	50.9 ± 6.5	50.9 ± 4.1	1
Left ventricular tele-diastolic volume (ml/m²)	54.6 ± 13.1	54.9 ± 11.5	0.93
Left ventricular ejection fraction	65.3 ± 7.4	65 ± 8.2	0.89
Left ventricular mass (g/m²)	91.2 ± 18.5	94.5 ± 17.2	0.5
Mitral regurgitation >grade 1	0 (0%)	0 (0%)	1
Maximal left atrial volume (ml/m²)	33.8 ± 10.4	36.6 ± 9.3	0.3
E (cm/s)	71.7 ± 18.1	77.3 ± 14.4	0.21
Septal e′ (cm/s)	8.7 ± 2.2	8.6 ± 2.1	0.87
Lateral e′ (cm/s)	10.6 ± 2.6	12.1 ± 3.1	0.06
Septal E/e′	8.8 ± 3.3	9.4 ± 2.5	0.42
Lateral E/e′	7 ± 2	6.7 ± 1.6	0.54
**E/A fusion**	**0 (0%)**	**1 (4%)**	**1**
**Data Exercise 25 Watts**			
Heart rate (bpm)	88.4 ± 10.5	82.6 ± 11.8	0.06
E (cm/s)	87.5 ± 18.1	92.6 ± 23.1	0.37
Septal e′ (cm/s)	10.1 ± 2	11 ± 2.4	0.14
Lateral e′ (cm/s)	12 ± 2.3	13.3 ± 3.4	0.11
Septal E/e′	9 ± 2.9	8.6 ± 1.7	0.54
Lateral E/e′	7.6 ± 2.2	7.2 ± 1.7	0.46
**E/A fusion**	**0 (0%)**	**3 (11%)**	**0.24**
**Data Exercise 50 Watts**			
Heart rate (bpm)	98.2 ± 12.3	93.1 ± 11.3	0.12
E (cm/s)	101.5 ± 22.8	105.6 ± 23.9	0.52
Septal e′ (cm/s)	11.8 ± 2.2	12.1 ± 2.3	0.63
Lateral e′ (cm/s)	14.1 ± 3	14.7 ± 3.6	0.51
Septal E/e′	8.8 ± 2.4	9 ± 2.2	0.75
Lateral E/e′	7.5 ± 2.2	7.5 ± 1.8	1
**E/A fusion**	**6 (22%)**	**3 (11%)**	**0.46**

Values are means ± SD or *n* (%).

### Impact of NID on the association between septal E/e′ and LVEDP

A multivariable regression analysis (ANCOVA, n = 54) was performed for determination of LVEDP _at rest/25/50 watts_ as a function of septal E/e′ value, NID use or not, presence of known coronaropathy, heart rate, and indexed left ventricular mass. Septal E/e′ rather than lateral E/e′ was chosen since correlations between septal E/e′ and LVEDP were stronger at rest, 25 and 50 watts (Table 4) than those of lateral E/e′ (Table 4).

**Table 4:** Correlation between LVEDP and echocardiographic surrogates for LVFP.

	All
Number of examinations	54
**Correlation with LVEDP**_**at rest**_	
Septal E/e'_0Watts_	**r = 0.35** *
Lateral E/e'_0Watts_	**r = 0.31** *
**Correlation with LVEDP**_**25Watts**_	
Septal E/e'_25Watts_	r = 0.41*
Lateral E/e'_25Watts_	r = 0.31*
**Correlation with LVEDP**_**50 Watts**_	
Septal E/e'_50 Watts_	r = 0.34*
Lateral E/e'_50 Watts_	r = 0.17

*Significant with p < 0.05.

At rest, only septal E/e′_at rest_, was significantly (β = 0.36, p = 0.007) associated with LVEDP _at rest_ without influence of NID. During exercise, at 25 and 50 watts, only septal E/e′_at 25/50 Watts_ (β = 0.43, β = 0.34; respectively, p ≤ 0.01) and NID use or not (β = −0.26, β = −0.28, respectively, p < 0.05) were significantly (p ≤ 0.01) associated with LVEDP _at 25/50 Watts_. The other parameters tested were not significantly associated to LVEDP value _at 25/50 Watts_.

**Figure 2:**
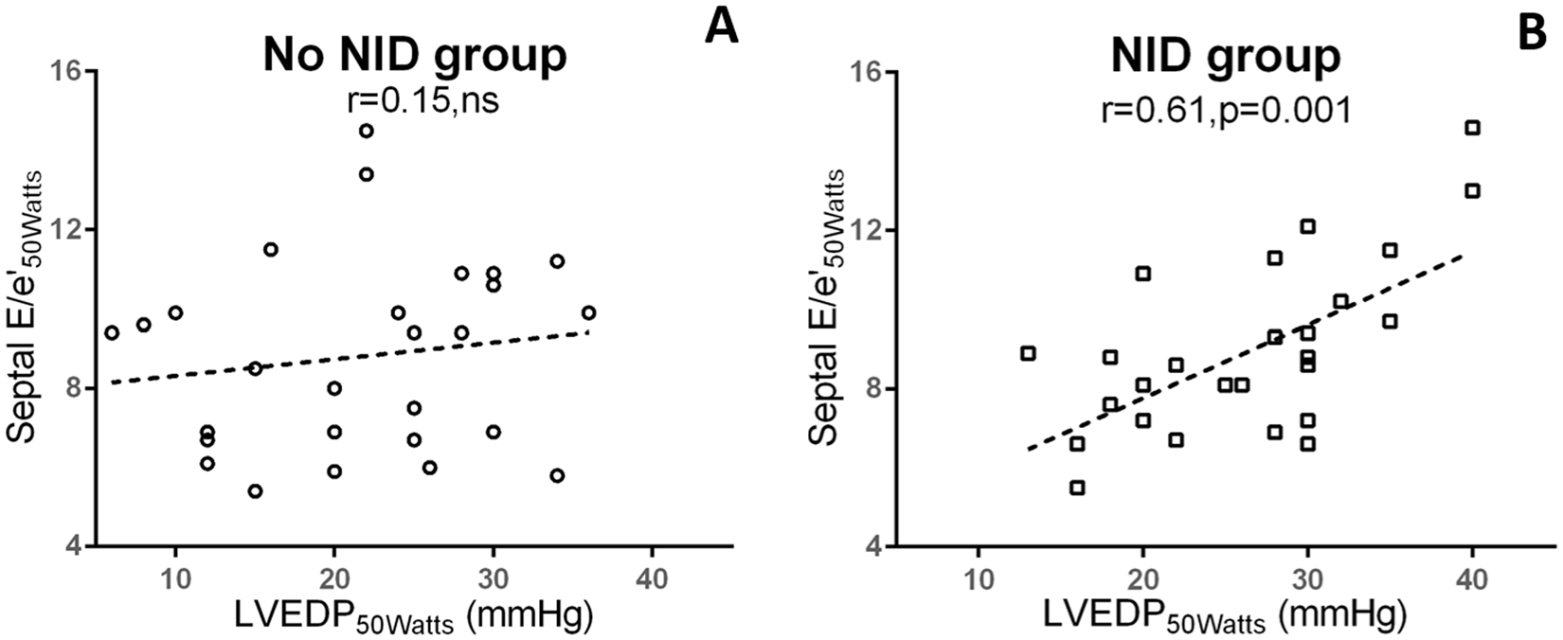
Correlation and linear regression between LVEDP and septal E/e′ at 50 Watts according to the absence (**A**) or presence of negative inotropic drug (NID) use (**B**).

Interestingly, in subgroups analysis, correlation between septal E/e′ _at 25 Watts_ and LVEDP _at 25 Watts_ was slightly better in NID group (r = 0.5, n = 27, p < 0.01) compared to no NID group (r = 0.43, n = 27, p < 0.05). At 50 watts, differences in the correlation between septal E/e′ _at 50 Watts_ and LVEDP _at 50 Watts_ were further improved with good correlation in patients on NID (r = 0.61, n = 27, p < 0.05) while it disappeared (r = 0.15, n = 27, ns) in the no NID group (Fig. 2).

In the group of patients on NID (n = 27), ROC curves analysis for prediction of abnormal stress LVEDP (>16mmHg and ≥25mmHg) from septal E/e′ _at 50 Watts_ showed an area under the curve of 0.8 (p = 0.09) and 0.72 (p ≤ 0.05), respectively. In the group of patients off NID (n = 27), ROC curves analysis for prediction of abnormal stress LVEDP (>16mmHg and ≥25mmHg) from septal E/e′ _at 50 Watts_ were not significant (area under the curve: 0.67 and 0.61, p = ns, respectively).

## Discussion

In this prospective study, we evaluated the impact of NID on the accuracy of Doppler echocardiography to assess LVFP in patients with preserved LVEF at risk of developing abnormal exercise LVFP increase at low workload levels as the reflection of early stage HFpEF. We found that NID use had no influence on the association of septal E/e′ with LVEDP at rest. During exercise, the correlation between septal E/e′ and LVEDP persisted only if patients were on NID. These results may explain the conflicting data about the validity of stress E/e′ for the assessment of exercise LVFP in patients with preserved LVEF. This finding may have a clinical significance particularly when considering that NID are frequently withdrawn in routine practice before a stress test.

### Controversy about validity of echocardiography for evaluating LVFP during exercise

Several studies found that E/e′ was a better surrogate for LVFP at rest in patients with low LVEF on beta-blockers compared to patients with preserved LVEF^5, 8, 24–26^. Later, it was found that this correlation between E/e′ and LVFP was abolished by catecholamine use both in patients with and without cardiac systolic dysfunction^21, 22, 27, 28^. In recent years, concerns have been raised about the reliability of echocardiography for estimating LVFP during exercise. A small sample size study found a good correlation between LVEDP and E/e′ in patients with variable LVEF, mostly on beta-blockers^3^. This finding was also confirmed in another study in patients with advanced HFpEF mostly on betablockers^14^. In contrast, several recent studies reported the absence of association between LVEDP and E/e′ or their variations during exercise, in untreated patients with preserved LVEF^11, 13^. In our relatively large and well phenotyped cohort, we found that NID use influences the association between stress E/e′ and the direct invasive measure of LV diastolic pressure as the reference method. Thus, our results give new insight concerning previous discordant results. Moreover, our study emphasizes the importance of NID maintenance for stress echocardiography when performed for diagnosis of HFpEF. Recommendations concerning beta-blockers management before stress echocardiography are lacking in the recently updated guidelines for the evaluation of left ventricular diastolic function by echocardiography^4^.

### Inotropic agents alter the e′ dependence to LVFP

It has been shown in animal models that positive inotropic drugs improve LV relaxation (shortening constant of relaxation Tau) and increase the dependency of e′ on load variations^18–20^. By contrast, e′ variation is little affected by filling pressure and has been validated as a preload-independent index of LV relaxation^17^ in cases of Tau lengthening, such as that associated with left ventricular systolic dysfunction^29^ or with NID use such as beta-blockers^18^. This may explain why use of E/e′ as a surrogate of LVFP has mostly been validated in patients with structural heart diseases, in particular LV systolic dysfunction on beta-blockers^25, 26^ and not in patients on positive inotropic drugs or during exercise. In fact, exercise is responsible of endogenous release of catecholamine, i.e. positive inotropes^23^. Therefore, our findings in humans confirm these preclinical findings in canine models where use of inotropes impaired the association between E/e′ and hemodynamic evaluation of LVFP, as opposed to beta-blockers, which improved it^18–20^.

### Limitations

Heart catheterization and trans-thoracic echocardiography were not performed at exactly the same time and this may have contributed to decrease the magnitude of correlations between LVEDP and E/e′. However, good quality echocardiographic exams are difficult to obtain in catheterization laboratories and can lead to acquisitions of poor quality. Moreover, this methodological choice ensured that echocardiographic acquisitions and analyses of the data were made blindly of invasive measurements. The patients were investigated using exactly the same exercise protocol and without any modification of their drug treatments.

In patients treated with NID, treatment strategies were highly heterogeneous, with various doses and types of NID used, potentially leading to different effects on heart and vascular function. Too few patients were included in this study for additional multivariable analyses to be performed to assess any influence of type and dose of NID. Of note, in current clinical practice, strictly identical echocardiographic parameters are used for the estimation of LVFP in patients with different type and doses of NID or either without NID.

Since the non-invasive diagnosis of early stage HFpEF remains a major clinical challenge, we decided to focus our investigations on patients with preserved LVEF. The assessment of the influence of NID on stress E/e′ diagnostic accuracy in patients with altered LVEF deserves further investigations. In these latter patients, a focus on several comprehensive evaluations after careful beta-blocker dose up-titration is needed, seeking for dose-effect impact of NID on the relationship between E/e′ and LVFP.

## Methods

### Study

This study was ancillary to the PREFFORT study (NCT01714752)^30^. The study protocol and all methods applied were approved by the Committee for the Protection of Human Subjects of Pitié-Salpétière (Paris, France) and prior written informed consent was obtained from all subjects after being fully informed regarding the nature and risks of the study. We did the study in accordance with the principles of the international conference on harmonization guidelines on good clinical practice and the world medical association declaration of Helsinki.

### Patient selection

The methods was previously described in details elsewhere^30^. Briefly, we prospectively enrolled 60 patients, among those admitted to the Pitié-Salpêtrière hospital in Paris, France, between 12/2012 and 07/2013, for clinically indicated left heart catheterization. Included patients had to undergo invasive and non-invasive assessments of LVFP within 24 hours, without change to their medications during that interval.

All included patients fulfilled the following criteria: (1) in sinus rhythm with LVEF >50%; (2) without any significant valve disease, hypertrophic cardiomyopathy, heart transplantation, atrial fibrillation, severe mitral annular calcification, LV thrombus, severe renal failure and (3) without any acute coronary syndrome in the previous 3 months or angiographic diagnosis of a stenosis requiring a percutaneous coronary intervention.

The risk factors of coronary artery disease, especially age, hypertension and diabetes, are also recognized as risk factors of HFpEF^2^. Thus, the patients referred for coronary angiography were also at high risk of developing abnormal exercise LVFP as the reflection of early stage HFpEF^2^.

### Clinical data

On admission, demographic data (age, sex, previous coronaropathy, diabetes, hypertension, other comorbid conditions), baseline clinical data, type of NID (non-dihydropyridine calcium channel blockers, beta-blockers) and of other current treatments were collected prospectively. The type and dose of NID were left at the discretion of the treating physician.

### Invasive hemodynamic assessment

After realization of the coronary angiography for diagnostic purpose, a fluid-filled pigtail catheter introduced through the radial artery was positioned in the mid-LV cavity using fluoroscopy, with the patient in the supine position. This catheter was connected to a strain-gauge pressure transducer positioned at right atrium level and zeroed before measurement. LV pressure tracings were recorded at baseline and during exercise. Measurements were made off-line, blindly from echocardiographic and clinical data by two experienced physicians. LVEDP measurement was averaged over five cardiac cycles. A hemodynamic LV end-diastolic pressure (LVEDP) ≤16 mmHg was defined as normal at rest. An increase of LVEDP >16 mmHg (moderate), and to a further extent ≥25 mmHg (important), was considered abnormal during stress^1, 30, 31^.

### Echocardiographic assessment

Transthoracic echocardiography was performed using a Vivid 9 (GE Healthcare; Horten, Norway) by an experienced physician blinded to clinical and invasive data. Images were transferred to a workstation equipped with the Echopac PC software version 12.0 (GE Vingmed Ultrasound®; Horten, Norway). All examinations were analyzed off-line. All measurements were averaged over 3 to 6 cardiac cycles.

The following measurements were performed according to current guidelines^32^: LV internal diameter, inter-ventricular septal and posterior wall thicknesses at end diastole from M-mode. LV mass was derived. Left atrial maximal volume was measured in 2-dimensional mode at the end of ventricular systole just before opening of the mitral valve by biplane Simpson method from apical four and two-chamber views. LV volumes and LVEF were measured using the biplane Simpson method. When appropriate, preceding measures were indexed to body surface area. Trans-mitral E and A were determined by pulsed-Doppler echography. Using tissue-Doppler data, we acquired e′ in early diastole, in spectral mode, for both the basal septal and lateral segments, using the four-chamber apical view (Fig. 1). The calculated variables were lateral E/e′ and septal E/e′. Intra and inter-observer variabilities of E/e′ measurements at rest and during exercise were excellent (intra-class correlation coefficient ≥0.94 and ≥0.86, respectively)^30^.

### Exercise Protocol

The same two-leg pedaling exercise was performed within 24 hours in catheterization laboratory using a cycle ergometer ERG 911 BP/XRAY (Schiller®, Switzerland) and then, in echocardiography laboratory using ERG 911 L/LS ergometer (Schiller®, Switzerland). Patients pedaled (50–60 cycles/minute) in the supine position, beginning at a workload of 25 Watts for 3 minutes, then at 50 Watts for another 3 minutes. Heart rate, blood pressure, invasive hemodynamic and non-invasive echocardiographic data were collected at baseline and during the last minute of each level of exercise, i.e. 25 and 50 Watts (Fig. 1). Continuous electrocardiographic monitoring was undertaken throughout the examination. The patient medications, including NID type and doses were unchanged between the invasive and non-invasive evaluations. Patients were asked at the end of the 50 Watts exercise if they had experienced dyspnea during the effort. Adequate mitral inflow, tissue Doppler signals and hemodynamic variables were obtained for all patients at baseline. E/e′ could not be measured accurately during exercise in two patients and hemodynamic data at exercise were missing in 4 patients, leaving 54 patients for final analysis.

### Statistical analysis

For quantitative variables, mean and standard deviations (SD) were calculated. Discrete variables are presented as percentages. Continuous variables were compared with unpaired *t* tests for normally distributed variables. Fischer’s exact test was used for categorical variables. Statistical significance was defined as *P* < 0.05. All tests were two-tailed. Repeated measure two-ways ANOVA with Sidak post-test were performed to compare changes in LVEDP between rest, 25 and 50 Watts, as a function of NID use or not. The correlation between variables was assessed by calculating Pearson’s or Spearman’s coefficient (r), as appropriate. Multiple regression analysis (ANCOVA) was used to study association between LVEDP and E/e′, considering other covariates such as NID use (XLStat, Addinsoft®), at rest, 25 and 50 Watts. The choice of using septal or lateral E/e′ for these analyses was determined by the strength of their respective correlation with LVEDP. Receiver-operator characteristics (ROC) curves were plotted to determine diagnostic properties of E/e′ value to predict exercise LVEDP >16 mmHg and ≥25 mmHg, as a function of NID use (GraphPad 6 Software, San Diego, USA).

## Conclusion

NID use is an important confounding factor to be taken into account when assessing exercise LVFP using stress E/e′ in patients with preserved LVEF.
